# Luciferase gene of a Caribbean fireworm (Syllidae) from Puerto Rico

**DOI:** 10.1038/s41598-019-49538-7

**Published:** 2019-09-10

**Authors:** Yasuo Mitani, Rie Yasuno, Ryo Futahashi, Todd H. Oakley, Yoshihiro Ohmiya

**Affiliations:** 1 Bioproduction Research Institute, National Institute of Advanced Industrial Science and Technology (AIST), Sapporo, 062-8517 Japan; 2 Biomedical Research Institute, AIST, Tsukuba, 305-8566 Japan; 3 Bioproduction Research Institute, AIST, Tsukuba, 305-8566 Japan; 4 Marine Science Institute, University of California, Santa Barbara, CA 93106 USA; 5 DAILAB, Biomedical Research Institute, AIST, Tsukuba, 305-8566 Japan

**Keywords:** Biogeography, Biogeography

## Abstract

The fireworms *Odontosyllis* spp. are globally distributed and well-known for their characteristic and fascinating mating behavior, with secreted mucus emitting bluish-green light. However, knowledge about the molecules involved in the light emission are still scarce. The fireworms are believed to emit light with a luciferin-luciferase reaction, but biochemical evidence of the luciferase is established for only one species living in Japan and no information is available for its luciferin structure. In this study, we identified a luciferase gene from a related Puerto Rican fireworm. We identified eight luciferase-like genes in this Puerto Rican fireworm, finding amino acid identities between Japanese and Puerto Rican luciferase-like genes to be less than 60%. We confirmed cross reactivity of extracts of the Japanese fireworm luciferin with a recombinant Puerto Rican luciferase (PR1). The emission spectrum of recombinant PR1 was similar to the crude extract of the native luciferase, suggesting that PR1 is a functional luciferase of this Puerto Rican fireworm. Our results indicate that the molecular mechanism of luminescence is widely conserved among fireworms.

## Introduction

A wide variety of luminous animals are distributed on the earth. Some of them are terrestrial, many are marine, while only one species is known from fresh water^1,2^. Since the first cloning of a luciferase gene involved in light emission from the firefly *Photinus pyralis*^3^, a diversity of luciferase genes has been reported, mainly from terrestrial organisms^2,4^. While terrestrial beetles basically use the same light emission system with D-luciferin as a substrate among different families, luminous marine animals use various luciferins including coelenterazine, *Cypridinid* luciferin, and dinoflagellate luciferin^2,5^.

The fireworms *Odontosyllis* spp., including *O*. *enopla*, *O*. *phosphorea*, *O*. *undecimdonta*, *O*. *octodentata*, and *O*. *luminosa*, are distributed sporadically in locations around the world including the Caribbean Sea, West Coast of the United States, and Japan^6–11^. Despite many observations of their characteristic mating behavior, the molecular mechanisms underlying light emission is not well understood^1,2^. Luciferin structure has not been determined although more than 50 years ago Shimomura *et al*. partially purified luciferin and luciferase from more than 50,000 individuals of *O*. *enopla*^12^.

Recently, we identified a fireworm luciferase gene using unpurified luminous mucus from the Japanese species, *Odontosyllis undecimdonta*^13,14^. This luciferase gene was independently identified by another group using protein purification procedures^15^. The recombinant luciferase protein produced the same characteristic bluish-green emission peak as the wild fireworms^14,15^. It should be noted that the fireworm luciferase gene has an evolutionarily different origin from other known luciferase genes because the fireworm system did not show any cross reactivity to known systems including coelenterazine and *Cypridinid* luciferin^14^. In a third study, transcriptome analyses of the Bermuda fireworm *O*. *enopla* identified luciferase-like genes^16^, although its biochemical function has not been confirmed.

In this study, we focused on a fireworm from Puerto Rico. A species living in Puerto Rico was originally described as *O*. *octodentata*^17^ with behavior similar to *O*. *enopla*^18^. Although we have not determined the species in detail, we confirmed the cross reaction between the recombinant luciferase of Japanese fireworm and the ethanol extract of a Puerto Rican fireworm which probably contains fireworm luciferin. RNA sequencing (RNA-Seq) and subsequent analyses identified 8 possible luciferase paralogous genes (PR1-PR8), among which PR1 shows the highest expression level. The recombinant PR1 exhibited light emitting activity in the presence of crude luciferin extract from the Japanese species. These data strongly suggested that the Japanese *O*. *undecimdonta* and the Puerto Rican species share molecular mechanisms of bioluminescence through luciferin-luciferase (LL) reaction.

## Results and Discussion

### Collection of the fireworm in Caribbean Sea and the LL reaction

**Figure 1:**
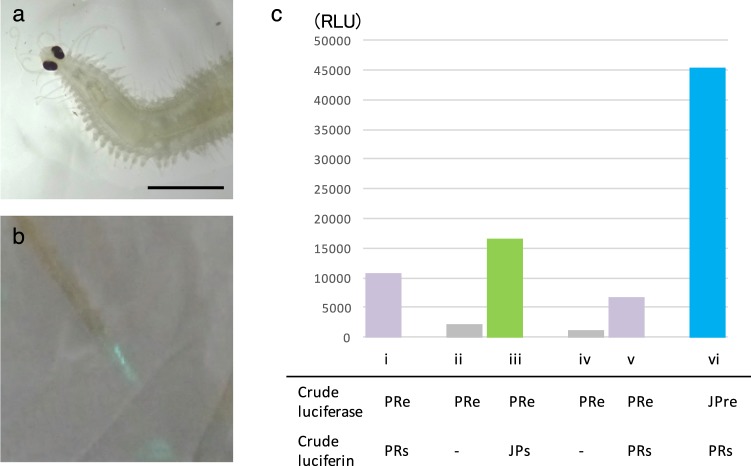
The Puerto Rican fireworm. (**a**) Bright field image of anterior part. (**b**) Luminous mucus from a worm that was pressed on paper. The bluish-green color appeared similar to that observed in their natural secretion during the mating behavior on the sea surface. Scale bar = 2 mm. (**c**) i to v are a series of assays using different combination of substrate extract (PRs and JPs) using the native PRe. A 10 μL of PRe was measured in 100 μL RB in total (i) and left for 3 hours (ii). Then, 1 μL JPs was added to this solution to check the cross reaction (iii). The activity went down to almost background level after 1 hour of the reaction (iv) and 10 μL PRs was added to the mixture (v). The activity using 1 μL JPre and 10 μL PRs is shown in (vi).

We collected the fireworm in the Caribbean Sea, off Puerto Rico (Fig. 1a). The fireworms came to the sea surface 30 to 40 min after sunset and disappeared suddenly approximately 30 min after the appearance of the first visible displays, as described previously^18^. Their appearance peaked a couple of days after the full moon, when more than one hundred females were observed to make luminous circles. Although it was not difficult to collect them, we could not keep them alive for long periods, even in a sea table with fresh seawater flowing. After they were collected, they did not emit light even after strong stimulation; however, when they were pressed against a paper towel, a similar bluish-green light as observed on the sea surface was confirmed (Fig. 1b). This feature was different from the Japanese species because the they emit strong light when stimulated by a needle tip^14^. This is probably because the Caribbean individuals already secreted most of their luciferase and luciferin outside the body as a mating signal.

To conduct cross-reactivity tests, a single specimen of the Caribbean fireworm was crushed to obtain crude luciferase extract (PRe) while another specimen was soaked in ethanol to obtain crude luciferin (PRs). We used some animals for the cross reaction tests, but it was difficult to obtain clear numerical data because their activities were different from worm to worm due to the residual level of luciferase and luciferin in the body. Thus, we presented typical cross reaction data in Fig. 1. The luminous activity of the luciferase extract was confirmed visually just after crushing (Fig. 1c,i). This extract was left in a refrigerator for 3 hours to consume the luciferin (Fig. 1c,ii). The addition of the crude luciferin obtained from *O*. *undecimdonta* (JPs) recovered the luminescent activity (Fig. 1c,iii). Then, this solution was left in a refrigerator for 1 hour to exhaust the added luciferin (Fig. 1c,iv). The addition of PRs recovered the luminescent activity (Fig. 1c,v). Finally, the light emission activity was confirmed for the mixture of recombinant luciferase of the Japanese fireworm *O*. *undecimdonta* (JPre) and PRs (Fig. 1c,vi). Thus, cross-reactions between these two species were confirmed. These results suggested that the light emission mechanisms underlining these two different fireworms are the same, although we were not able to obtain quantitative data due to difficulties of collecting enough active animals.

### Luciferase homolog of a fireworm from Puerto Rico

**Figure 2:**
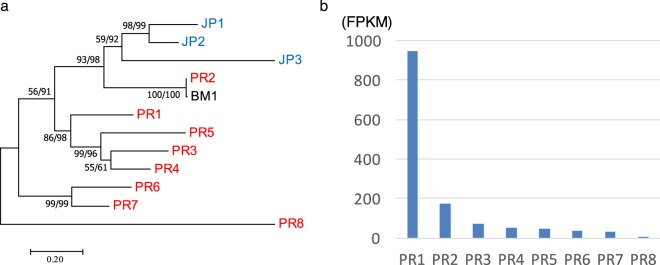
Molecular phylogeny and expression analysis of the Caribbean fireworm luciferase paralogs. (**a**) Molecular phylogenetic analysis of fireworm luciferases. A maximum likelihood phylogeny is shown, while neighbor-joining phylogeny exhibit the same topologies. Statistical supporting values are indicated on each node in the order of (bootstrap value of maximum likelihood)/(bootstrap value of neighbor-joining). (**b**) Relative expression level of eight luciferase-like gene in whole body of single individual calculated based on RNA-Seq analysis.

**Figure 3:**
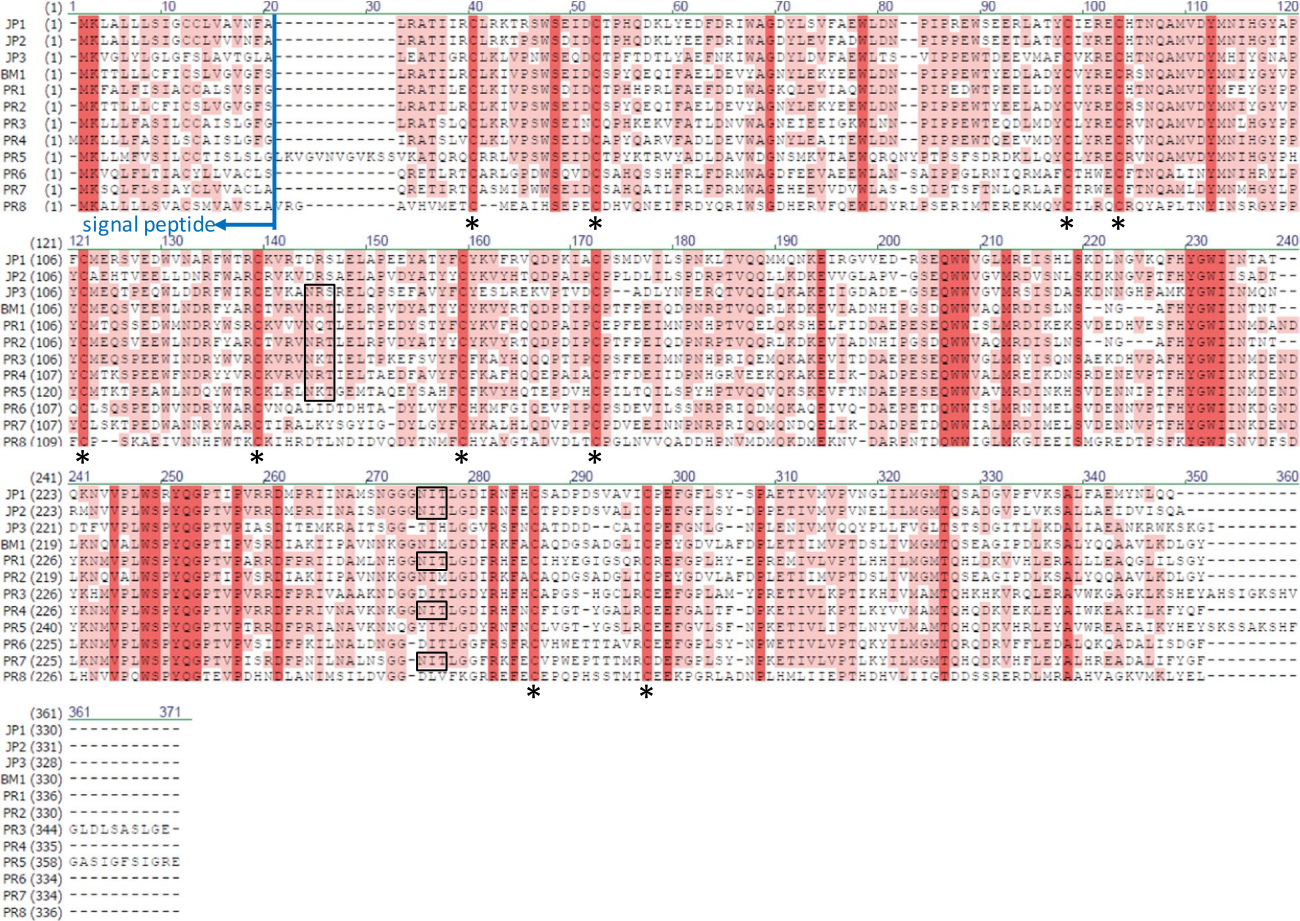
Comparison of amino acid sequences among fireworm luciferase-like genes. JP, BM, and PR mean Japanese *O*. *undecimdonta* (Mitani *et al*.^14^), Bermuda *O*. *enopla* (Brugler *et al*.^16^), and Puerto Rican (this study) genes, respectively. Identical or conserved amino acid residues are shaded by red and pink, respectively. *N*-terminal secretion signal sequence is indicated by arrows. *N*-glycosylation motifs are indicated by boxes. Conserved cysteine residues are indicated by asterisks.

To obtain the luciferase genes, we performed RNA-Seq analysis using a single individual of Puerto Rican fireworm to exclude genetic diversity derived from polymorphism. After manual assembly, we identified 8 possible paralogous genes (PR1-PR8) with significant sequence similarity to Japanese and Bermuda fireworm luciferase genes (Fig. 2a). We numbered the genes in descending order of expression level with PR1 as the gene with the highest expression (Fig. 2b). From the molecular phylogenetic tree, three paralogous genes from the Japanese fireworm luciferase formed a single clade with the Puerto Rican fireworm luciferase PR2 and Bermuda fireworm luciferase BM1 together forming a sister clade. This molecular phylogenetic tree indicates that the luciferase gene duplication occurred independently in Japanese and Puerto Rican fireworms. The identity of amino acids between Japanese fireworm luciferase and Puerto Rican fireworm luciferase was less than 60% (Table 1), but the positions of 10 cysteine residues in all luciferase-like genes were conserved (Fig. 3). The Bermuda fireworm luciferase-like gene (BM1) was almost identical to PR2 with only a single amino acid substitution (99% identities). The high similarity of these genes suggests our Puerto Rican species is very similar or the same species as that analyzed previously from Bermuda. Although several luciferase-like paralogs were reported in the Bermuda fireworm^16^, there was no biochemical data of their activity as luciferase. All these luciferase-like proteins contained probable secretion signal peptides at each N-terminus (Fig. 3). As shown for *Cypridina* luciferase^19,20^, at least one *N*-glycosylation motif characteristic of secreted proteins was found in 10 genes of 12 fireworm luciferase-like genes except PR6 and PR8 (Fig. 3, boxes).

**Table 1:** Percent of identities, positives, and gaps between Japanese and Puerto Rican fireworm luciferase homologues.

	Identities	Positives	Gaps
JP1 vs PR1	184/330 (56%)	228/330 (69%)	3/330 (0.01%)

The number of identical amino acid residues and those with similar properties are shown as Identities and Positives, respectively.

### Recombinant PR1 protein expression and its secretion property

**Figure 4:**
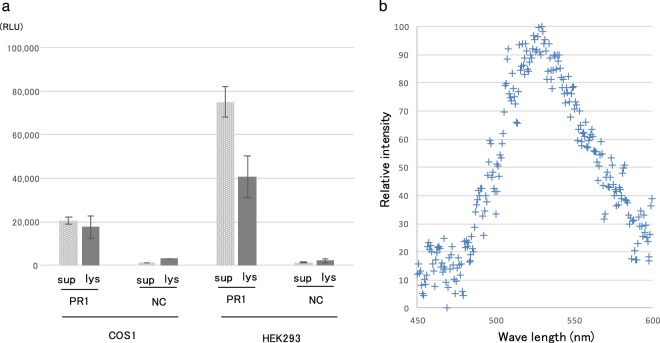
Recombinant luciferase expression using mammalian cells COS1 and HEK293. (**a**) Expression vector containing luciferase gene PR1 or empty vector as negative control (NC) was expressed and their activities were monitored. In each case, supernatant (sup) and lysate (lys) were analyzed to detect secretion and cytosolic recombinant protein, respectively. (**b**) Spectrum analysis of a mixture of recombinant PR1 protein and crude luciferin extract.

In order to clarify the function of a Puerto Rican fireworm luciferase-like gene, we synthesized recombinant protein of PR1 which showed the highest expression level among Puerto Rican luciferase-like genes in Fig. 2. We produced recombinant PR1 by mammalian expression systems using COS1 and HEK293 cells. Luciferase activity was measured separately for supernatant of culture media (Fig. 4a, sup) and whole cell lysate (Fig. 4a, lys) after collection of the cells by centrifugation, because recombinant protein was expected to be secreted into the culture media. The recombinant protein exhibited significant light emitting activity for both supernatant and lysate fractions compared to negative control (Fig. 4a), and the ratio of the secretion fraction was higher than that of Japanese fireworm luciferase JP1 (Table 2)^14^. Importantly, like the Japanese fireworm luciferase^14^, the emission spectrum of recombinant PR1 was similar to the native luciferase crude extract with a possible peak around 510–530 nm (Fig. 4b), suggesting that PR1 is a functional luciferase gene. Improvement of recombinant PR1 protein production is necessary to obtain a clear emission spectrum to clarify its emission peak. Unfortunately, we were not able to obtain the emission spectrum using native luciferin extract from the Caribbean species due to low amount of samples.

**Table 2:** Secretion rate of the recombinant luciferases.

Host cell	Clone	Secretion rate (Std. dv.)/%
COS1	JP1	0.918 (0.238)
	PR1	41.7 (9.54)
HEK293	JP1	18.4 (3.09)
	PR1	33.9 (4.14)

Estimated secretion rate is shown with standard deviation (std. dv.) of three independent experiments. COS1 and HEK293 were used as host cells and JP1 and PR1 were expressed.

## Methods

### Animal collection

Fireworms were collected in La Parguera (17.95N, 67.05W), Puerto Rico on July. 22–29, 2016. The fireworm length ranged from 5 to 30 mm. Animals were kept frozen for a long-term storage or soaked in RNAlater (Thermo Fisher Scientific) solution and kept frozen for RNA extraction.

### LL reaction

Fifty worms of the Japanese species were put into 1.5 mL of 99.5% ethanol, and the supernatant after short centrifugation was used as the crude luciferin solution (JPs). A single specimen of the Caribbean species was crushed in 100 μL of the reaction buffer (RB) containing 50 mM Tris-HCl (pH 7.5), 300 mM NaCl, and 20 mM MgCl_2_ and the supernatant collected after centrifugation at 20,000 × g for 10 min was used as a crude luciferase solution (PRe) while another specimen was soaked in 100 μL of ethanol and the supernatant was used as a crude luciferin extract (PRs). The recombinant luciferase for *O*. *undecimdonta* (JPre) was described previously^14^. In the light emission activity assays, normally 1 μL JPs, 1 μL JPre, 10 μL PRe, and 10 μL PRs were used with an appropriate combination in 100 μL RB in total. The activity was monitored using luminometer, Phelios AB-2350 (ATTO) and recorded as relative light unit (RLU) for 10 s accumulation of the measurement. Emission spectrum was measured using a high sensitivity CCD spectrophotometer, AB-1850S (ATTO).

### RNA-Seq and gene analysis

Total RNA was extracted from one whole individual using Maxwell 16 LEV Simply RNA Tissue kit (Promega). RNA-Seq analysis was performed as described previously^14,21^. cDNA libraries were constructed using TruSeq RNA Sample Preparation Kits v2 (Illumina) and sequenced by MiSeq (Illumina). The raw reads were subjected to de novo assembly by using Trinity^22^ implemented in the MASER pipeline^23^. After automatic assembling, we checked and manually corrected the putative luciferase sequences as reported previously^24^. After manual assembly, sequence read mapping was performed using the BWA-mem software^25^ implemented in the MASER pipeline, whereby the transcript expression levels were estimated to calculate the fragments per kilobase of exon per million (FPKM) values. Signal peptide sequences were predicted by using SignalP^26^. Multiple alignment was performed using ClustalW (DNA Data Bank of Japan). Protein similarity was estimated using BlastP program (NCBI) to find identical and similar character amino acid sequences. To construct the molecular phylogeny of fireworm luciferase genes, deduced amino-acid sequences were aligned using the Clustal W program implemented in MEGA7^27^. Molecular phylogenetic analyses were conducted by the neighbor-joining method and the maximum-likelihood method using MEGA7. Bootstrap values for neighbor-joining and maximum likelihood phylogenies were obtained by 1000 resampling. Maximum Composite Likelihood model and Tamura Nei model were used for neighbor-joining and maximum likelihood analysis, respectively^28^. Bootstrap values for neighbor-joining and maximum likelihood phylogenies were obtained by 1000 bootstrap replications.

### Recombinant protein expression in mammalian cells

Gene sequence coding PR1 optimized to human codon usage was synthesized and inserted into a pcDNA3.1- vector by an outsourcing company (Genscript). COS1 cells were cultured in Dulbecco’s modified Eagle’s medium (DMEM, Wako) supplemented with 10% fetal bovine serum (FBS) at 37 °C in a humidified incubator exposed to 5% CO_2_. COS1 cells were transfected with expression plasmids using Lipofectamine 3000 (Invitrogen). On the following day, the culture medium was replaced with serum-free DMEM. The culture medium was collected 24 h after medium exchange and used as the supernatant fraction (sup). Cells were lysed with 10 mM Tris-HCl (pH 7.4) using sonication. The lysate was centrifuged at 20,000 × g for 5 min, and the resulting supernatant was used as the cell extract fraction (lys). The activity of recombinant PR1 in each fraction was measured by adding NaCl and MgCl_2_ solutions to a final concentration of 300 mM and 20 mM each, and adding crude luciferin extract obtained from *O*. *undecimdonta*^14^. The luminescence of the mixture was immediately measured using a Phelios AB-2350 luminometer (ATTO). Secretion ratio was determined as the activity ratio of sup to total (sup plus lys) activity.
